# The Legal Aspect of Madness

**Published:** 1918-09-21

**Authors:** 


### PAPERS ON MADNESS.

### V. The Legal Aspect of Madness.

It is because madness is mainly and primarily disorder of conduct that it differs from every other disease in having necessarily a legal aspect. That which affects one man alone and has no bearing on the welfare of his neighbours is of no concern to the law, and no law has any bearing upon it. The only exception to this rule is the act of suicide, and the law takes cognisance of suicide for two reasons; first because it is an act. It is a part of conduct, and the law applies to conduct and to conduct alone. No law regulates what a man may think or feel or desire or will, until his mental process issues in conduct. No law prescribes what is to go on in his mind any more than it prescribes what is to go on in his body. The law does not attempt to regulate a man's delusions, or any other disorder of his mind, any more than it attempts to regulate his digestion or his circulation, or any disorder in his liver or lungs. But the moment a man affects the welfare of his neighbours, at that moment the law steps in with its prohibition or its admonition, and prescribes what he shall or shall not *do*. It determines, in short, what his conduct is to be. The law takes account of nothing but conduct. The law seeks to regulate nothing but conduct, and even of conduct, that part only that affects the welfare of others.

The only exception is that already noted, of suicide, and with respect to the legal prohibition of suicide there are two things to be said. In the first place, it is in its origin no part of the civil law. It is a part of the ecclesiastical law which has for some reason become embodied in the civil law, or rather, which, in the separation of ecclesiastical from civil law has remained on the wrong side of the dividing line. Originally in this country there was no distinction between the civil law and the ecclesiastical law. The bishop sat side by side with the justiciar, and shared with him not only the judgment seat, but the control of the proceedings and the interpretation or proclamation of the law; and the law administered in the courts was a medley of civil and ecclesiastical law. In the natural course of evolution, the functions of the bishop and the judge became separated. The bishop retired to his own court, over which he alone presided, and left the judge presiding alone over the civil court; but even then the separation of civil and ecclesiastical offences was not complete. Certain offenders were triable in either court, or indeed in both; and an acquittal in one left him still in danger of conviction in the other; and when the separation became complete the line of division was not in every case logically or properly drawn. Certain civil offences, such as slander and adultery, remained triable in the ecclesiastical courts: certain ecclesiastical offences, such as those which are the subjects of the Criminal Law Amendment Acts and suicide, offences against morality but not against the fabric of society, remained triable in the civil courts.

The civil law takes cognisance of nothing but conduct, but it does not apply to all parts of conduct, nor ought it to do so. The ecclesiastical law takes cognisance of certain departments of conduct with which the civil law does not concern itself; and the ecclesiastical law goes farther, and embraces in its purview not only a man's deeds and words, but also his thoughts. It pries into the secret recesses of his mind, and calls him to account for his thoughts, his feelings, his desires. Those alienists who still regard madness as a malady of the mind alone, and refuse to recognise that what gives it all its terrors, all its inconveniences, all its drawbacks, all its difficulties, is not disorder of mind, but disorder of conduct, those belated and benighted alienists who still take this view are still in the shackles of mediævalism. They are pervaded by the ecclesiastical spirit. They are still but one stage removed from the doctrine that madness is demoniacal possession. It is true that they are but few, and that their influence is small, but it still lingers in places, and especially in London. The doctrine is a survival in medicine of a bygone state of things, and is comparable to the survival in law of the Norman-French proclamation "Oyez, Oyez," with which our courts open their proceedings. It is interesting to the historian, but it is of no practical moment to the alienist physician of the present day.

The civil law takes cognisance of conduct alone, and of conduct only in as far as it affects the welfare of other people. No law prohibits a man from suffering from scarlet fever or small-pox, but the law rightly prohibits him from doing that which may communicate his scarlet fever or small-pox to others; and when the law does interfere with him, what it interferes with is what he does; it interferes with his action, with his conduct. It does not say You shall not suffer this; it says You shall not do this. The reason the madman becomes, as such, amenable to the law, the reason the law takes immediate cognisance of him, is because of what he does, not because of what he thinks, or feels, or desires. And even of what he does the law need take no account, and for ages did take no account, unless what he does affects injuriously the welfare of others. The harmless lunatic was until recent years outside the purview and scope of the law, and if he has recently come within the cognisance of the law it is for his own protection. It is because the law has taken a new view of its duty, and now seeks to protect each member of society, not only from the depredations of others, but also from the harm that he may inflict upon himself, or may suffer, on account of his incapacity—of his incapacity to act, and not merely to think, or feel, or desire in a normal manner.

☛ Previous articles appeared on March 2, August 24 and 31, and Sept. 14, page 515.

**Papers on Madness**—(*continued*).

The original ground that justifies and requires the cognisance of the madman by the law is that the conduct of the madman either harms or threatens the welfare of others; but a secondary ground is that the madman must be restrained from harming himself, either in person or in property. Then as he must be restrained from doing harm, he is apt to be restrained excessively, and to suffer injustice and wrong. He must be restrained; but that he may be restrained no more than is necessary, those who keep him in restraint must be made responsible in law for his treatment. This is one chief purpose of the law relating to lunatics. Another, which looms far larger in the estimation of the public, though the experience of the last seventy years shows that their fears are greatly exaggerated, and almost wholly unwarranted, is to secure that no one who is not actually mad shall be improperly restrained under the pretence or under the mistake that he is mad. Further, the restraint that it is necessary to impose upon a madman must always include, and is sometimes restricted to, restraint from dealing with his property. Hence the law of lunacy naturally falls under several headings.

First, the law places on certain bodies the duty, and allows to certain other bodies and individuals the permission, to exercise restraint upon madmen, and to house, feed, clothe, and care for them. Second, the law provides that these restraining bodies and persons shall be responsible to the Government that the exercise of their powers shall be sufficient and no more than sufficient. Third, in order that no person who is not in fact mad shall be restrained as a madman, the law provides certain formalities to be observed and certain documents to be signed before a lunatic can be restrained; and as an additional safeguard, more than one person must concur in every case before restraint can be exercised. Lastly, there is an elaborate code of law regulating the manner in which the estate of the madman is to be administered for his benefit. With the exception of those provisions of the criminal law that relate to madmen and madness, the whole of the Law of Lunacy is combined under these four heads.

Let us again emphasise, first that the madman is restrained solely on account of his tendency to do harm to others or to himself in person or property or feelings; and second, that he is restrained on account of his tendency to *do* harm, not to think or wish or will harm. It is his conduct, and his conduct alone, that justifies the step. The law will not interfere with any man for evil thinking alone, but if he expresses his evil thoughts in evil words or evil acts, at once he is obnoxious to the law. Neither will the law interfere with him for evil wishing or evil willing. It is action and action alone that matters. We have no business and no right to judge a man mad for anything that he thinks or feels, but only for what he says and does. As long as his conduct is sane, he is a sane man, though his mind may be as full of delusions as a herring is of little bones; and if his conduct is mad, he is mad, whether we can find delusion in his mind or not.

